# Low-Power Chemiresistive Gas Sensors for Transformer Fault Diagnosis

**DOI:** 10.3390/molecules29194625

**Published:** 2024-09-29

**Authors:** Haixia Mei, Jingyi Peng, Dongdong Xu, Tao Wang

**Affiliations:** 1 Key Lab Intelligent Rehabil & Barrier free Disable (Ministry of Education), Changchun University, Changchun 130022, China; 17826070895@139.com; 2 Shanghai Key Laboratory of Intelligent Sensing and Detection Technology, School of Mechanical and Power Engineering, East China University of Science and Technology, Shanghai 200237, China

**Keywords:** dissolved gas analysis, chemiresistive gas sensors, transformer fault diagnosis, low power, cross-sensitivity

## Abstract

Dissolved gas analysis (DGA) is considered to be the most convenient and effective approach for transformer fault diagnosis. Due to their excellent performance and development potential, chemiresistive gas sensors are anticipated to supersede the traditional gas chromatography analysis in the dissolved gas analysis of transformers. However, their high operating temperature and high power consumption restrict their deployment in battery-powered devices. This review examines the underlying principles of chemiresistive gas sensors. It comprehensively summarizes recent advances in low-power gas sensors for the detection of dissolved fault characteristic gases (H_2_, C_2_H_2_, CH_4_, C_2_H_6_, C_2_H_4_, CO, and CO_2_). Emphasis is placed on the synthesis methods of sensitive materials and their properties. The investigations have yielded substantial experimental data, indicating that adjusting the particle size and morphology structure of the sensitive materials and combining them with noble metal doping are the principal methods for enhancing the sensitivity performance and reducing the power consumption of chemiresistive gas sensors. Additionally, strategies to overcome the significant challenge of cross-sensitivity encountered in applications are provided. Finally, the future development direction of chemiresistive gas sensors for DGA is envisioned, offering guidance for developing and applying novel gas-sensitive sensors in transformer fault diagnosis.

## 1. Introduction

Oil-immersed power transformers are critical components in the operation of power grids. The line voltage must be increased or decreased both at the power station and the consumer end. Therefore, the reliable operation of oil-immersed power transformers is a critical factor in ensuring the safety and stability of the power system [1]. Transformer oil serves as a coolant and plays an essential role in protecting the insulation during transformer operation. Under the influence of electricity and heat, transformer oil gradually decomposes, producing gases such as H_2_, CH_4_, and C_2_H_2_, which dissolve in the oil. Early diagnosis and identification of transformer faults can be achieved by analyzing the composition, concentration, and correlation of these dissolved gases [2]. The dissolved gas analysis (DGA) technique is recognized as the most convenient and effective method for diagnosing transformer faults [3].

Transformer insulating oil is a mixture of different hydrocarbon molecules containing CH_3_*, CH_2_*, and CH* groups linked by C-C bonds. Electrical or thermal faults can break certain C-H and C-C bonds, resulting in the generation of active hydrogen atoms and unstable free radicals of hydrocarbons, which can be recombined through complex chemical reactions to form H_2_ and lower hydrocarbon gases (CH_4_, C_2_H_6_, C_2_H_4_, and C_2_H_2_). Low energy discharges, such as partial discharges contribute to the breaking of the weakest C-H bond (338 KJ/mol) by ionic reactions, which accumulate mainly by recombination to H_2_. Higher temperatures (more energy) are required for the breaking of the C-C bond, which is then rapidly resynthesized into hydrocarbon gases in the form of the C-C bond (607 KJ/mol), the C = C bond (720 KJ/mol), and the C≡C bond (960 KJ/mol), requiring higher temperatures and higher energies, in that order. CH_4_ and C_2_H_6_ are typically produced at 500 °C, while C_2_H_2_ is typically produced at temperature ranging from 800 °C to 1200 °C, so large quantities of C_2_H_2_ are produced in the arc path [4].

In addition, solid insulation materials such as paper, laminates, or wood blocks contain a large number of anhydrous glucose rings in their molecules, as well as weak C-O and glucose pincer bonds, which can be re-decomposed at lower temperatures. The effective temperature for polymer cleavage is above 105 °C, and complete cleavage and carbonization occur at temperatures above 300 °C, producing large amounts of CO and CO_2_, as well as water.

Additionally, solid insulation materials such as paper, laminates, or wood blocks contain a large number of anhydrous glucose rings, weak C-O bonds, and glucose pincer bonds within their molecules, which can recombine or re-decompose at lower temperatures. The effective temperature for polymer cleavage is above 105 °C, while complete cleavage and carbonization occur at temperatures higher than 300 °C, producing large amounts of CO, CO_2_, and water.

To summarize the above points, the primary and secondary characteristic gases produced by the different fault types can be summarized in Table 1, and the gase concentration range for multi-component gases online monitoring device [5] is shown in Table 2.

The sampling of dissolved gases in transformer oil can be divided into oil sampling and gas sampling. Transformer oil is always obtained from the tank valve of the monitored equipment through the oil sampling part. Then, the oil and gas separator is used to achieve the separation of the dissolved gases from the transformer oil. For gas sampling, the gases can be collected directly at the gas relay vent nozzle if the fault gases are collected inside the gas relay.

Gas-sensing detection technology is the core of DGA technology, directly influencing the timeliness, accuracy, and reliability of transformer fault diagnosis. Gas chromatography and new gas sensors are at the forefront of research and application in dissolved gas detection technology for transformer oil. While using gas chromatography to analyze the dissolved gases in the oil is a well-established method for monitoring the oil-filled electrical equipment, it has several drawbacks. These include expensive detection equipment, complicated operation, and reliability issues, such as detection insensitivity and inaccuracy. Consequently, there is a growing demand for the development of gas detection devices with high reliability and low maintenance to ensure the safe and stable operation of power grids.

New gas sensors represent the future direction of transformer fault detection technology. Researchers have explored various types of gas sensors for DGA technology, including infrared spectroscopy [6], Raman spectroscopy [7], photoacoustic spectroscopy [8], laser calorimetry spectroscopy [9], quartz crystal microbalance sensors [10], tunable diode laser absorption spectrometry [11], catalytic combustion sensors [12], and chemiresistive sensors [13,14,15,16]. Among these diverse types, chemiresistive gas sensors have garnered widespread attention due to their high sensitivity, broad detection spectrum, long lifetime, low cost, and simple structure. Additionally, the all-solid-state nature of these materials enables potential miniaturization, intelligent functionality, and integrated development, making them highly attractive to researchers.

Since the invention of the first chemiresistive gas sensor, called the Taguchi Gas Sensor, by Figaro Corporation in 1968, Metal Oxide Semiconductor (MOS) gas sensors have continuously attracted the attention of both the industry and the market [17]. However, for the inherent characteristics of semiconductors, traditional gas sensors require high operating temperatures to exhibit good gas-sensing performance. High operating temperatures increase heating power consumption, device size, and cost, limiting the application of these sensors in battery-powered portable devices and failing to meet the growing demand for IoT end devices [18]. Considerable research has been devoted to the development of high-performance and low-power gas sensors [19,20,21]. The emergence of MOS-based MEMS gas sensors in the early 1990s provided a new low-power gas sensor strategy [22], which will likely dominate the low-power gas sensor market [23,24,25]. In addition, the development of high-performance cryogenic materials has received much attention as another direct and effective way to reduce gas sensor heating power consumption. Some typical 1D or 2D materials have been widely used as gas-sensing materials. Examples include CNTS [26], graphene [27], Mxenes [28], TMD [29], etc. These efforts will bring the complex DGA technology closer to portable, real-time monitoring.

In this review, the sensitivity mechanism of chemiresistive gas sensors is analyzed, and the latest research results on low-power gas sensors designed for detecting dissolved fault characteristic gases (H_2_, C_2_H_2_, CH_4_, C_2_H_6_, C_2_H_4_, CO, and CO_2_) are presented. Their material synthesis methods and sensitivity properties are also highlighted. The main approaches to enhancing the sensitivity characteristics of chemiresistive gas sensors and reducing their power consumption are summarized, as well as strategies to address the critical issue of cross-sensitization. Finally, the future development of chemiresistive gas sensors for transformer fault detection is envisioned.

## 2. Sensitivity Mechanism for Chemiresistive Gas Sensors

### 2.1. Adsorbed Oxygen Theory

As the most typical chemiresistive gas-sensing material, MOS is based on the principle that various chemical reactions between the sensing material and the gas molecule cause a change in the resistance parameter. Changes in the nature and concentration of the target gas are thus converted into a corresponding electrical signal.

Typically, semiconductors can be categorized into n-type semiconductors and p-type semiconductors. The charge carriers in intrinsic semiconductors can be controlled by the appropriate doping of donors or acceptors. The main carriers in broadband oxide semiconductors are determined by the stoichiometry of the doped covalent cations or oxygen vacancies. For example, undoped oxygen-deficient SnO_2_ exhibits n-type semiconductivity because of electron generation and the formation of oxygen vacancies. The p-type semiconductivity of undoped NiO can be explained by the absence of metal ions in the material [30].

MOS generally behaves as an insulator at room temperature. At 100–500 °C, the electrons of oxygen molecules adsorbed on n-type oxide semiconductors (e.g., SnO_2_ and ZnO) are trapped due to the trapping capability of the semiconductor material’s surface, resulting in changes in the adsorbed oxygen species [31]. For n-type semiconductors, an electron depletion layer in a high-resistance state will eventually form in the semiconductor surface, while an n-type semiconductor region in a low-resistance state will appear in the inner core. On the other hand, for p-type semiconductors, a hole accumulation layer in a low-resistance state will eventually build up on the semiconductor surface, while an insulating center in a high-resistance state will arise in the inner core. The root of the gas-sensitive properties of MOS is the redox reaction between the target gas and the oxygen adsorbed on the semiconductor surface, which ultimately leads to a change in material resistance. At different temperatures, the type of oxygen adsorbed on the material surface varies, and the chemical mechanism of the reaction also changes.

### 2.2. Charge Transfer Mechanism

For some typical 1D or 2D materials, such as MXenes, carbon-based materials, e.g., their sensitive properties depend mainly on the charge transfer mechanism, i.e., the gas molecules are physically adsorbed on the material through van der Waals or donor–acceptor interactions [32]. The more adsorption sites there are on the sensitive material, or the higher the binding energy between the sensitive material and the target gases, the better it is for the detection of the gas molecules. Surface charge transfer is usually achieved by direct carrier exchange between the adsorbed gas and the nanomaterial. When gas molecules are exposed to the surface of nanomaterials, the oxidizing gas molecules (e.g., NO_2_) tend to extract electrons from the nanomaterial, while the reducing gas molecules (e.g., NH_3_) provide electrons to the nanomaterial. For n-type nanomaterials, the conductivity typically decreases when the sensing layer is exposed to oxidizing gas molecules. Unlike oxidizing gases, reducing gases increase the conductivity of the n-type sensing layer. For p-type nanomaterials, oxidizing and reducing gases produce opposite changes in conductivity.

## 3. Novel Contributions

### 3.1. H_2_ Gas Sensors

H_2_ is one of the critical characteristic gases for transformer fault diagnosis. Currently, most of the research and applications of H_2_ sensors are focused on gas leakage detection with a high limit of detection (LOD) (the explosion limit of H_2_ is 4%) [33]. In comparison, the H_2_ LOD for DGA is lower, accompanied by the high heating power consumption of semiconductor gas sensors [34,35], so the development of high-performance and low-power H_2_ sensors is one of the hot spots of domestic and international research [36,37].

By investigating a series of research works, it was found that the performance of sensors can be significantly improved by doping the noble metal Pd into the conventional semiconductor materials SnO_2_ [38,39], ZnO [40], WO_3_ [41], and In_2_O_3_ [42]. This is because H_2_ molecules are small enough to cleave into the interior of the Pd lattice, where they combine with Pd atoms to form hydrides (PdHx). The surface palladium hydride (PdHx) has a lower figure of merit than pure palladium, accelerating the transfer of free electrons from the metal palladium to the surface of the MOS and leading to improved sensor performance. By adjusting the particle size, morphology, and specific surface area of the traditional semiconductor material, combined with the noble metal Pd doping, low temperature and low LOD of H_2_ can be realized to satisfy the requirements of H_2_ detection in DGA technology.

Duan et al. [43] prepared MOF-derived MOS nanocomposites for H_2_ sensing at room temperature by combining the advantages of noble metal nanoparticles and photoexcitation. It was found that H_2_ could be fully recognized by the original electrical signal of the sensor, as 5.0Pd@ZnO exhibited a typical p-type semiconductor response to H_2_ and a typical n-type semiconductor response to the other combustible gases (CO, MeOH, EtOH, and H_2_S). Meanwhile, the prepared sensors combine the advantages of MOF and MOS materials with high linear response, good repeatability, and long-term stability.

MEMS sensors, which can realize low power consumption, miniaturization and integration of gas sensors, could be the mainstream of future gas sensors. However, for MEMS sensors, one of the key technologies is the precise loading of the sensing material onto the specified micro-region. Chen et al. [44] propose a new method for the preparation of MEMS gas sensors. The process guides the spontaneous flow of Pd doping into mesoporous In_2_O_3_, modified for the hydrophilic sensing region by pre-growing patterned self-assembled monolayers with superhydrophobic properties in the non-sensing region of the microchip. The fabricated MEMS gas sensors have an ultrahigh response to H_2_ gases with concentrations in the range of 0.5–100 ppm.

**Figure 1:**
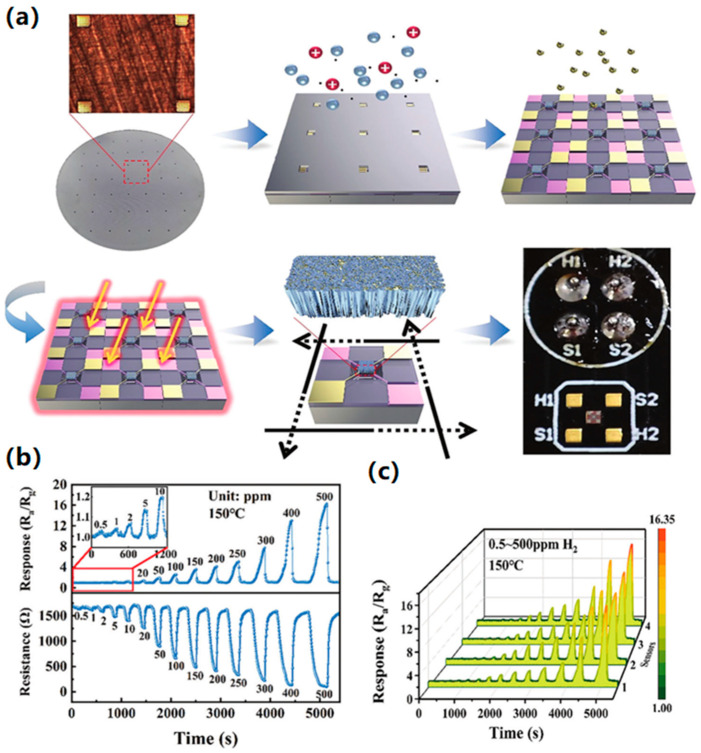
MEMS H_2_ sensor [45]. (**a**) Wafer-level fabrication process. (**b**) Response curve. (**c**) Consistency testing.

The preparation of uniform films at the wafer level is an essential prerequisite for realizing the high consistency of the sensing chip in the mass production of MEMS gas-sensitive chips. Commonly, methods such as drop-coating and screen printing cannot fulfill this requirement due to low precision and uncontrollable technology. At the same time, these methods easily lead to contact between the sensing material and the electrode pads, resulting in signal crosstalk between the heated electrode and the test electrode, even affecting the effectiveness of the subsequent packaging. In addition, noble metal catalysts are useful for improving gas sensitivity and selectivity. However, the modification of noble metals is usually prepared by solution impregnation on the surface of powdered materials, which is challenging to realize in wafer-level fabrication processes. To address these process issues, Zhang et al. developed a simple method combining atomic layer deposition and magnetron sputtering to experiment with wafer-level production of MEMS H_2_ sensors. As shown in Figure 1, the prepared MEMS H_2_ sensing chip shows a wide detection range, from 0.5 to 500 ppm, high resolution, and good reproducibility [45].

### 3.2. CH_4_ Gas Sensors

CH_4_ is the main characteristic gas for transformer oil or paper overheating fault determination. The molecular structure of CH_4_ is highly symmetric, and the C-H bond energy is as high as 439 kJ·mol^−1^, so CH_4_ shows strong chemical inertia. As CH_4_ is the main component of natural gas, it is used worldwide as a fuel for power generation and heating, and CH_4_ sensors have been developed early and are widely used for gas leakage detection. Meanwhile, CH_4_ is a greenhouse gas. Therefore, researchers have carried out a series of studies to detect CH_4_ under low power consumption [46,47,48,49,50].

**Figure 2:**
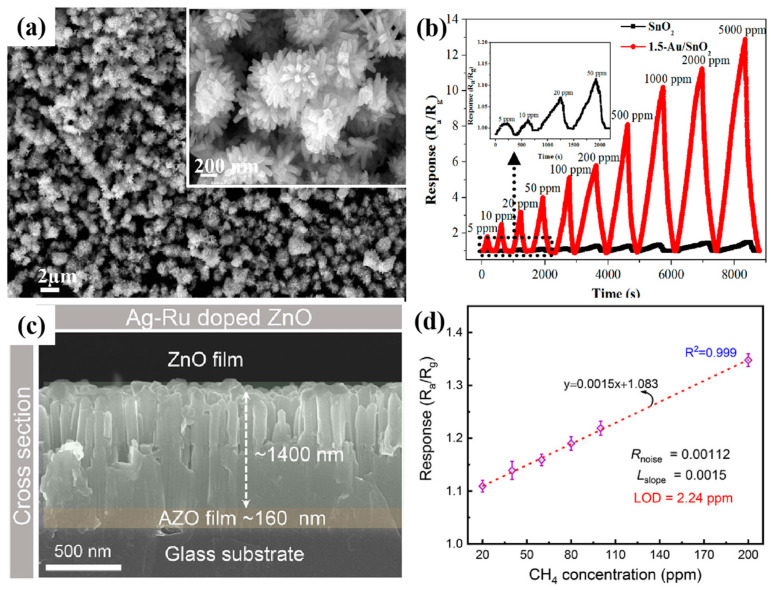
MOS for CH_4_ sensing. (**a**) SEM image of Au/SnO_2_ nanoflower. (**b**) Response of CH_4_ sensors to different concentrations of target gas (5–5000 ppm) [51]. (**c**) Ag-Ru co-doped self-assembled ZnO nanorods arrays. (**d**) The LOD fitting curve [52].

**Figure 3:**
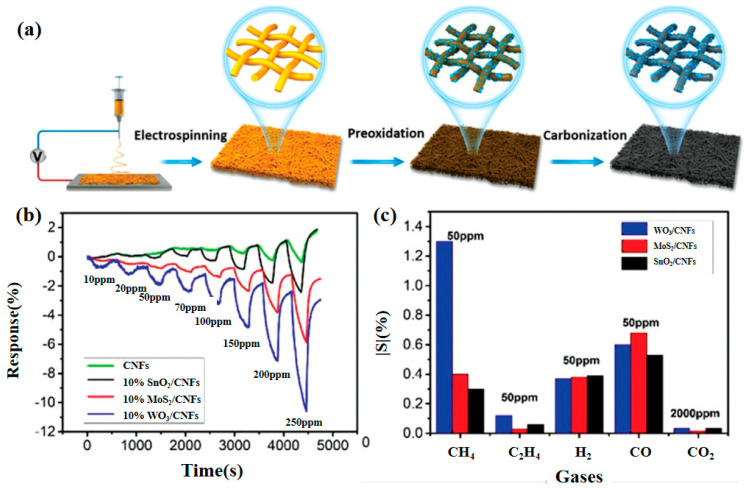
Three-dimensional mosaic membranes for CH_4_ sensing. (**a**) Mosaic film fabrication schematic. (**b**) Real-time response curves to 10–250 ppm CH_4_. (**c**) Cross−sensitivity [53].

Xue et al. [51] prepared Au-modified SnO_2_ nanoflower composites by a simple impregnation method and investigated their gas-sensitive performance for CH_4_, as shown in Figure 2a,b. The results showed that the sensor exhibited a good response to 5–5000 ppm CH_4_, with a LOD of 5 ppm. The excellent gas-sensing performance was mainly attributed to the structure of the nanoflowers and the catalytic properties of Au. Li et al. [52] prepared Ag-Ru co-doped self-assembled ZnO nanorod arrays by one-step hydrothermal, and their properties are shown in Figure 2c,d. The unique microstructure gives the composites good hydrophobicity, providing high moisture resistance. The oxygen vacancy density increases significantly after Ag-Ru co-doping. The Density Functional Theory (DFT) calculations showed that the charge density of the Ru sites was abnormally high, forming a localized strong electric field, which provided additional energy for the CH_4_ reaction at the surface-adsorbed oxygen–oxygen center point at room temperature. The optimized AgRu_0_._025_-ZnO exhibits excellent CH_4_ sensing performance with a LOD of 2.24 ppm under free-heat and free-light conditions. These findings suggest that the introduction of defects into the ZnO lattice, such as oxygen vacancies and localized ions, is effective in improving the gas-sensitive performance. Xu et al. [53] proposed the preparation of 3D mosaic membranes based on WO_3_, SnO_2_, MoS_2,_ and CNFs scaffolds by combining conventional inorganic semiconductor materials with carbon nanofibers, as shown in Figure 3. Due to the large specific surface area of the 3D network, as well as the synergistic and heterojunction effects between the composites, these three sensors exhibited a high response to CH_4_ at room temperature. This study provides a practical and scalable solution for the preparation of room-temperature chemiresistive sensors for gases such as CH_4_.

### 3.3. C_2_H_2_ Gas Sensors

C_2_H_2_ is the characteristic gas for transformer spark discharge and arc fault diagnosis. Since C_2_H_2_ is one of the important raw materials for organic synthesis, C_2_H_2_ sensors are currently mainly applied to leak detection of C_2_H_2_ with relatively mature technology [54,55,56]. The semiconductor materials used for detecting C_2_H_2_ are abundant and diverse, including typical SnO_2_ [57], ZnO [58], and WO_3_ [59]. However, the low sensitivity of intrinsic semiconductor materials cannot meet the practical application requirements for low-concentration detection. When the size of the semiconductor material is reduced to the nanometer level, the sensitivity performance of the material can be enhanced due to the increased specific surface area, thus enabling the detection of low concentrations of C_2_H_2_. Therefore, new structured semiconductor-sensitive materials have been continuously developed to meet the application requirements for C_2_H_2_ detection [60,61,62,63,64].

**Figure 4:**
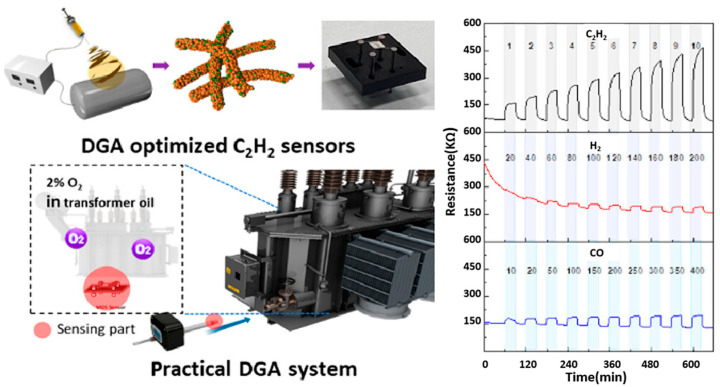
Highly sensitive and selective C_2_H_2_ sensor based on CuO/ZnO heterostructure for transformer diagnostics [66].

By controlling the type and amount of doped metal cations, the concentration of carriers and the number of surface dangling bonds in the sensitive materials can be regulated, thus improving the adsorption and reaction properties of gas molecules. Zhu et al. [65] reported the substitution of transition metals (TMs), such as Sc, Ti, V, Cr, Mn, Fe, Co, Ni, Cu, and Zn, at the b-site and n-site in boron nitride nanotubes (BNNTs), to improve the adsorption performance of the intrinsic BNNTs for oil-soluble gases (H_2_, CH_4_ and C_2_H_2_). DFT calculations showed that the adsorption properties of BNNTs for C_2_H_2_ gas are selectively enhanced due to the intervention of TM atoms. Jung et al. [66] developed a high-performance C_2_H_2_ gas sensor for DGA by constructing a CuO/ZnO heterostructure using an electrostatic spinning process. The CuO:ZnO = 8:2 sensor had the best performance, with a sensitivity of 7.6 for 10 ppm C_2_H_2_ and good stability in an analog transformer environment (see Figure 4). In addition, the sensor also showed high selectivity for transformer oil gases where H_2_, CH_4_, C_2_H_4_, C_2_H_6_, CO, and CO_2_ coexist.

**Figure 5:**
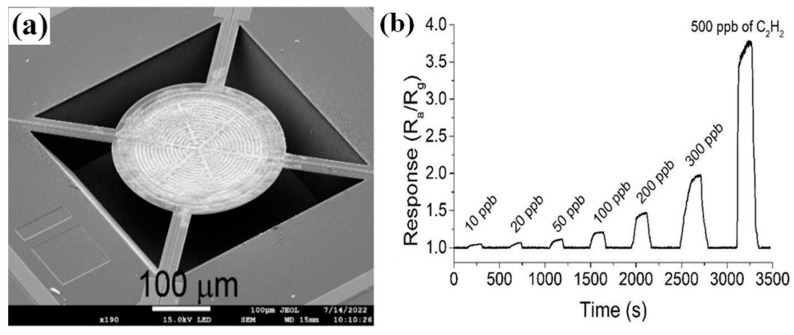
MEMS C_2_H_2_ sensor (**a**) SEM image of the micro hot plate (**b**) Response curve to 10–500 ppb C_2_H_2_ [67].

MEMS gas sensors have attracted much attention due to their low-power consumption characteristics. Xu et al. prepared a high-performance MEMS C_2_H_2_ sensor by using In_2_O_3_ nanoparticles obtained by co-precipitation as the sensing material [67], loading core-shell PtAg nanoparticles on the material surface as a catalyst, and combining a micro-hotplate chip as the electrode substrate. Due to the high catalytic performance of the core-shell-structured PtAg bimetallic nanoparticles, the sensor showed a high response to acetylene gas, with a detection limit of 10 ppb, as shown in Figure 5. The power consumption of the sensor was about 30 mW.

Two-dimensional materials have been used in the detection of C_2_H_2_ in recent years as emerging advanced functional nanomaterials due to their excellent chemical, physical, and electrical properties. Mohamed Shaban et al. [68] prepared nanoporous graphene oxide thin film sensors by combining the modified Hummer method with the spray pyrolysis technique. Their sensitivity properties to CO_2_, H_2_, and C_2_H_2_ at room temperature were investigated. The room temperature sensitivity of nanoporous graphene oxide can be attributed to the abundant hydroxyl groups and the nanopores on the surface of the material. In addition, the small band gap is another key to enabling the sensor to operate at room temperature based on van der Waals forces (physical adsorption), giving the nanoporous graphene oxide film a fast response and recovery properties at room temperature. Gui et al. [69] developed a ZnO/MXene sensor capable of detecting trace amounts of C_2_H_2_ at room temperature. The response–recovery rate was 14 s/6 s for 5 ppm C_2_H_2_, with a response rate of 178% and long-term stability of more than 30 days. In addition, the sensor was highly selective for C_2_H_2_.

### 3.4. C_2_H_4_ Gas Sensors

As the simplest olefin, C_2_H_4_ is an essential characteristic gas for determining overheating faults in transformer oil or paper. It is also a gas associated with environmental pollution, plant metabolites, and human health, which have received a great deal of attention [70,71,72,73,74,75].

**Figure 6:**
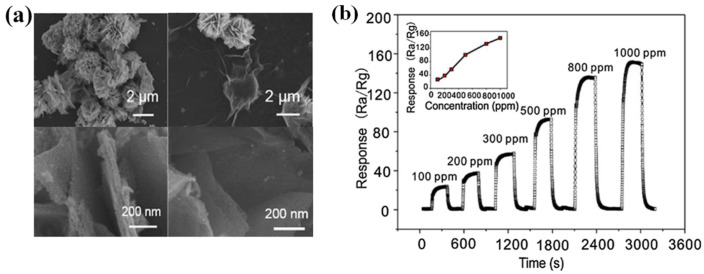
Pd/RGO/α-Fe_2_O_3_ for C_2_H_4_ sensing (**a**) SEM image. (**b**) Real-time response curve of the sensor [76].

The development of a feasible low-concentration C_2_H_4_ detection method is essential for the detection of dissolved gases in transformer oil. The surface and edges of graphene produced by the redox method usually contain a large number of structural defects, such as functional groups and lattice defects, which can be used for the improvement of sensor performance. Liu et al. [76] successfully synthesized flower-like porous α-Fe_2_O_3_ materials modified by Pd nanoparticles and RGO (see Figure 6). The flower-like hierarchical porous structure improves the specific surface area of the material. The catalytic effect of Pd nanoparticles and the chemically active defect sites of RGO synergistically improve the sensitivity performance of the material. The response recovery times of the fabricated sensors are 18 s and 50 s, with detection ranges from 10 ppb to 1000 ppm, respectively.

Gui et al. [77] proposed the preparation of Au-modified MoSe_2_ and its clusters for the adsorption of C_2_H_4_ gas molecules, which is one of the typical decomposition products of oil paper in transformers. Based on DFT, the adsorption system was constructed and analyzed using adsorption parameters and molecular orbitals. The results show that Au-3-MoSe_2_ can be used as a C_2_H_4_ adsorbent and sensing material for the detection and diagnosis of discharge faults in oil-immersed transformers.

### 3.5. C_2_H_6_ Gas Sensors

C_2_H_6_ is not a major gas component in transformer fault detection, but it is still a characteristic gas. C_2_H_6_ is one of the major components of gas, and sensor applications for gas detection have been popularized. However, there are fewer reports on the detection of C_2_H_6_, a saturated alkane, at low concentrations [78,79].

**Figure 7:**
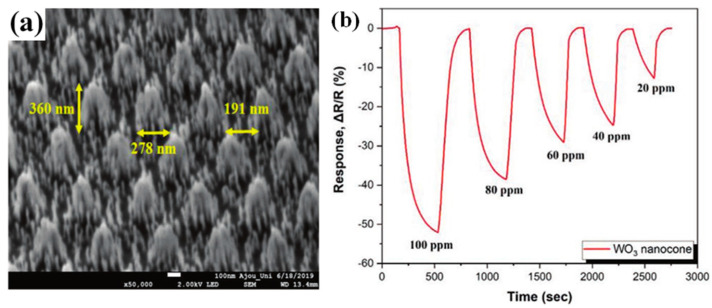
Three-dimensional WO_3_ nanocone for C_2_H_6_ sensing [80] (**a**) Microstructure of WO_3_ nanocone. (**b**) Response–recovery curve of the sensor for 100~20 ppm C_2_H_6._

Lee et al. [80] achieved the low-concentration detection of C_2_H_6_ gas by modulating the highly ordered 3D structure of a monolayer of WO_3_ in an isolation layer prepared by plasma-enhanced chemical vapor deposition and reactive ion etching with RF sputtering. Among them, the WO_3_-based gas sensor can reach 51.9% sensitivity for 100 ppm C_2_H_6_. Figure 7 shows the microstructure of WO_3_ nanocone and the response–recovery curve of the sensor for 100–20 ppm C_2_H_6_.

Qian et al. [81] used DFT to study the adsorption of C_2_H6 and C_2_H_4_ molecules on intrinsic and gold-doped MoS_2_ monolayers. The adsorption mechanism of molybdenum disulfide-based monolayers was investigated in terms of adsorption energy, adsorption distance, band gap structure, charge transfer, and the density of states. The computational results show that Au-doped MoS_2_ monolayers have larger adsorption energies, shorter adsorption distances, a higher charge transfer, and stronger orbital hybridization for C_2_H_6_ and C_2_H_4_ molecules compared to the corresponding MoS_2_ monolayer adsorption structures. This study provides theoretical guidance for the application of Au-MoS_2_ materials as C_2_H_6_ and C_2_H_4_ gas-sensing materials.

### 3.6. CO Gas Sensors

CO is the main component in the decomposition gas of transformer oil, and it is also one of the characteristic gases for transformer fault diagnostics. Currently, most of the research and application of CO sensors are aimed at air pollution. The U.S. Occupational Safety and Health Administration stipulates that the permissible exposure limit of CO within 8 h is 50 ppm, which is not much different from the lower limit of 25 ppm of dissolved gas concentration in transformer oil. Since CO pollution has been a long-standing concern, CO-sensing technology is relatively mature. Current research efforts are generally focused on improving the performance of CO sensors [82,83,84,85,86,87].

**Figure 8:**
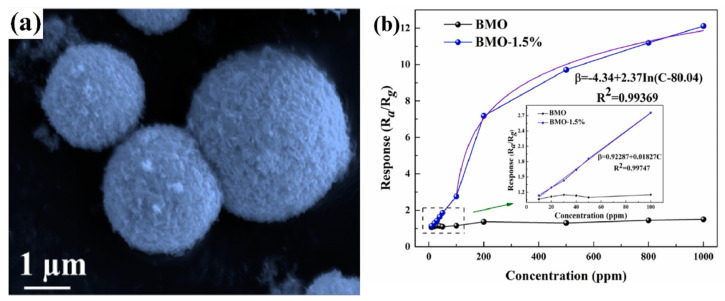
(**a**) SEM image of BMO–1.5% microspheres. (**b**) Fitted curves of response values of different concentrations of CO [88].

The bimetallic nanoparticle modification of polymetallic oxides is considered an attractive approach to improve the sensing performance. Zhang et al. [88] synthesized bimetallic PtPd-modified Bi_2_MoO_6_ microspheres by ultrasonication, rapid reduction, and solvent–thermal methods and then fabricated the sensor. The sensor exhibited excellent performance for 7.18–200 ppm CO, as shown in Figure 8. In practical applications, a good linear relationship is important for predicting unknown CO concentrations. The relationship between concentration C and the BMO–1.5% sensor response value β was: β = 0.92287 + 0.01827 C (R^2^ = 0.99747) over the 10–100 ppm concentration range. When the CO concentration is above 100 ppm, the relationship between C (100–1000 ppm) and the BMO–1.5% sensor response value β was: β = –4.34 + 2.37ln(C − 80.04) (R^2^ = 0.99369), where R^2^ denotes the fitness of the equation. The significant enhancement of the gas-sensitive performance of the sensor is attributed to the strong metal-carrier interaction and the sensitization of the noble metals Pt and Pd. This work provides a viable strategy for the analysis of the sensing mechanism.

**Figure 9:**
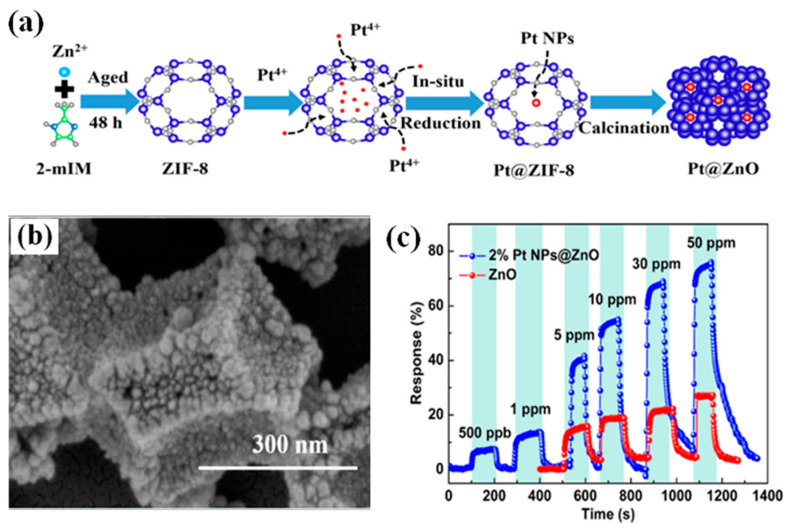
(**a**) Schematic of fabricated Pt @ZnO polyhedra. (**b**) SEM image of 2% Pt@ZnO polyhedra. (**c**) Response of 2% Pt @ZnO to different CO concentrations in 3.7 CO_2_ gas sensors [89].

Reducing the detection limit of MOS gas sensors by synthesizing porous nanostructures with a large number of active sites has also been shown to be effective. Wang et al. [89] used a metal-organic framework (MOF) (ZIF-8) as a template to cast highly dispersed Pt nanoparticles in situ on the pores of porous ZnO to form Pt NPs@ZnO polyhedral materials (see Figure 9). Compared with the pure ZnO material, the Pt NPs@ZnO polyhedral material has a higher response to CO gas. The prepared sensors have a good CO response and an ultra-low detection limit of 100 ppb. The high specific surface area, porous nanostructure, and electron sensitization effect are the main reasons for the enhanced CO-sensing performance of the Pt NPs@ZnO sensors, which have a ppb-level detection limit and a great potential for the detection of low concentrations of CO.

MOS-based gas sensors hold great promise for effective CO detection. However, improving sensor response and selectivity under humid conditions remains a key priority. Xia et al. [90] developed Pt quantum dot-modified MoS_2_ nanosheet (MoS_2_/Pt) composites. Promoted by visible light as a highly sensitive material for CO detection, the MoS_2_/Pt sensor exhibited a significantly improved response (87.4%) with impressive response–recovery kinetics (20 s/17 s), long-term stability (60 days), and good selectivity for CO at high humidity (nearly 60%). As a result, the MoS_2_/Pt surface promotes a CO response and selectivity, providing fundamental clues to improve gas detection in room-temperature semiconductor sensors under extreme conditions.

### 3.7. CO_2_ Gas Sensors

The traditional techniques for CO_2_ measurement are optical methods, including gas chromatography, infrared spectroscopy, and fluorescence. Despite the excellent sensitivity and selectivity of these methods for CO_2_ detection, they are bulky and expensive, and the development of inexpensive and miniaturized CO_2_ sensors has attracted great interest. However, due to the chemical inertness of CO_2_, there are fewer related studies [91,92,93,94].

Carbon nanotubes (CNTSs) are the most typical 1D room-temperature gas-sensitive materials. Due to their outstanding mechanical and electrical properties, carbon nanotubes have attracted more and more attention. Saad et al. [95] used spray pyrolysis to prepare thin films of ZnO and ZnO/CNTs and then investigated their response to CO_2_. The sensitivities of the ZnO and ZnO/CNTs sensors were 6.8% and 22.4%, respectively, at a CO_2_ flow rate of 150 sccm. The ZnO/CNTs sensor had a very stable sensitivity to CO_2_ for 21 days. In addition, the sensor had a high selectivity for CO_2_ compared to other gases, where the ZnO/CNTs sensor had a higher sensitivity for CO_2_ than for H_2_ and C_2_H_2_.

**Figure 10:**
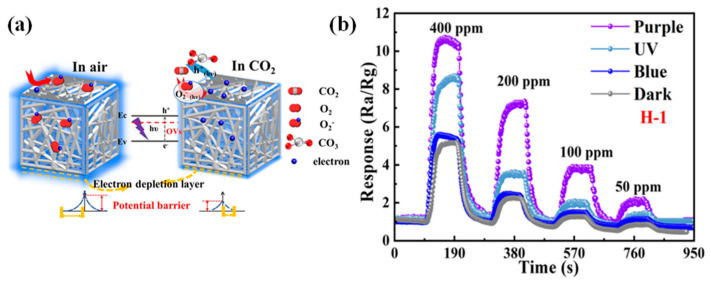
(**a**) Schematic diagram of the sensing process of ZnSnO_3_ exposed to air and CO_2_ gas. (**b**) Response–recovery curves of the sensor to 400–50 ppm CO_2_ under different light conditions [96].

Different surface morphology, surface area, and crystallinity of the sensitive materials are valuable for the design of room-temperature operable CO_2_ gas sensors. Zhang et al. [96] constructed 3D ZnSnO_3_ nanomaterials enriched with defective oxygen vacancies by adjusting the time of H_2_ treatment during the synthesis process, which, combined with visible-light excitation, achieved an ultra-low detection lower limit of 3.05 ppm for CO_2_. Figure 10a shows the schematic diagram of the sensing process of ZnSnO_3_ exposed to air and CO_2_ gas. Figure 10b shows the response–recovery curves of the sensor to 400–50 ppm CO_2_ under different light conditions. Shinde et al. [97] obtained Bi_2_O_3_ materials with varying areas of surface and structures by direct and ultrafast chemical bath deposition and applied them to CO_2_ detection. The performance and response–recovery time measurements of Bi_2_O_3_ nanosensors with different physical properties were superior to other target gases (H_2_, H_2_S, NO_2_, SO_2_) for CO_2_ gas. Among the different sensor morphologies, the nanosheet-type Bi_2_O_3_ sensor showed a response of 179% at a CO_2_ gas concentration of 100 ppm, with a response time of 132 s and a recovery time of 82 s at room temperature, which was attributed to its unique surface morphology, high surface area, and minimal charge transfer resistance. Soltabayev et al. [98] used the RF magnetron sputtering technique to prepare ZnO and Co_3_O_4_ powders as a single target for the deposition of Co-doped ZnO thin films on glass substrates. The changes in crystallinity, morphology, optical properties, and chemical composition of the films were investigated at sputtering powers of 45, 60, and 75 W. The films were characterized by the presence of a high concentration of CO_2_ and a high concentration of Co_3_O_4_. The response of CZO-60 W to CO_2_ was 1.45 at a CO_2_ concentration of 500 ppm, with response–recovery times of 72 s and 35 s. The distinctive feature of this sensor is that it is capable of detecting CO_2_ gas effectively and rapidly at room temperature.

## 4. Sensitization Methods

Gas-sensitive materials usually need to operate under certain temperature conditions to exhibit good gas-sensing properties. The gas-sensing mechanism is quite complex despite its simple working principle. The sensing performance, especially the sensitivity, is controlled by three factors: receptor function, transducer function, and utility [99]. The gas-sensing phenomenon occurs as a result of the coupling of these three factors. Therefore, one of the keys to achieving low-power gas detection is how to modify the sensitive material to compensate for the negative impact of low temperature on its performance.

### 4.1. Nanostructure Design

The nanostructural design of sensitive materials, such as high-energy surface exposure, can improve the reactivity of sensitive materials. The modulation of morphology can enhance the specific surface area of materials and increase the number of gases adsorbed, as well as improve the diffusion and transport rate of gases. Defect effects can provide more active sites for oxygen adsorption and gas reaction. A large amount of research data show that the response of sensitive materials to gases can be significantly improved by nanostructure design.

According to the nanoscale, sensitive materials can be classified into 0D (quantum dots [100], nanoparticles [101,102]), 1D (nanowires [103], nanorods [104,105], nanofibers [106]), 2D (nanosheets [107]), and 3D (nanoflowers [51]) materials.

Zero-dimensional materials mainly include quantum dots and nanoparticles. Quantum dots, which can also be referred to as nanocrystals, are widely studied in the field of gases. Quantum dots typically have diameters between 2 and 20 nanometers. High surface-to-volume ratios, edge and quantum confinement effects, and active edge sites make quantum dot materials highly sensitive gas-sensitive layers [34,35]. One-dimensional nanomaterials are materials with two dimensions within the nanometer length scale. One-dimensional nanomaterials are suitable gas-sensitive materials due to their large specific surface area, high porosity, and unique electronic properties. Currently reported sensing materials for dissolved gas detection in transformer oil are oxide semiconductors in various forms (nanorods [108], nanowires [109], nanofibers [66], etc., nanotubes [110]) and carbon nanotubes [111]. Two-dimensional materials have emerged as advanced functional nanomaterials in recent years due to their excellent chemical, physical, and electrical properties. The 2D materials used for the detection of dissolved gas in transformer oil can be engineered into nanostructures with different geometries, such as nanosheets [107]. In addition to this, novel materials such as graphene and its derivatives, such as graphene oxide (GO) and reduced graphene oxide (rGO) [112], have also been reported for the detection of dissolved gas in transformer oil. Since some low-dimensional nanomaterials are prone to aggregation, it is difficult to maintain good dispersion and structural stability, resulting in limited sensitivity, response speed, and poor reproducibility in gas detection. Three-dimensional nanostructures can overcome this obstacle and enhance gas-sensitive performance [113]. Three-dimensional nanomaterials have better pore and mechanical properties compared to 1D and 2D nanomaterials, and their structures are highly tunable, so they can be used as gas-sensitive materials to take advantage of the structural advantages and improve the activity of the materials.

### 4.2. Metal Cation Doping

Metal cations can be introduced into the semiconductor lattice as either donors or acceptors. By controlling the type and amount of doped metal cations, the concentration of carriers and the number of surface dangling bonds in the sensitive material can be regulated, improving the adsorption and reaction properties of gas molecules [114,115,116,117]. Jirasak Sukunta et al. [118] prepared Fe-doped SnO_2_ nanoparticles with different concentrations by the flame spraying method, investigated the sensitivity properties of the target gas C_2_H_2_ and explored the role of metal cation Fe in the sensing mechanism. Structural characterization demonstrated that Fe^3+^ in the nanoparticles formed a solid solution with the SnO_2_ lattice. The generation of chemisorbed oxygen on the surface of SnO_2_ and the formation of electron-withdrawal layers when the metal cation Fe was not doped resulted in the n-type semiconductor properties of the sensitive materials. With the increase in the Fe doping concentration, the Sn^4+^ ions in the lattice are replaced by Fe^3+^ ions, the lattice defects lead to the generation of holes, the space charge region is further expanded, the material resistance is subsequently elevated, and the material sensitivity performance is also enhanced and reaches its peak when the Fe doping amount is 0.1 wt%. In contrast, excessive Fe doping concentration increases the surface state density of SnO_2_ and decreases the material sensitivity.

### 4.3. Noble Metal Doping

Noble metal (Au, Pt, Pd, etc.) doping also contributes to the gas sensitization performance of chemiresistive gas sensors [100,101,102]. The introduction of noble metal catalysts can significantly reduce the activation energy of the reaction, which not only leads to an elevated response to the target gas but also improves the rate of the reaction, resulting in a shorter response–recovery time [119]. The noble metals’ action mechanism is now mainly explained from the perspectives of electron sensitization and chemical sensitization. Electron sensitization mainly refers to the formation of noble metal oxides when the noble metals are partially oxidized and act as electron acceptors to capture the electrons of the host material and form depletion layers at the material interfaces. When exposed to a reducing gas environment, the electrons are injected back into the host material from the noble metal oxide, thus achieving the effect of enhancing the sensitive response to the target gas. Chemical sensitization mainly refers to the “overflow effect” of the noble metal monomers, i.e., the noble metal dissociates the oxygen-negative ions, and the oxygen-negative ions overflow and adsorb onto the surface of the material, which increases the activity of the material and reduces the activation energy of the reaction to promote the adsorption and reaction of the gas molecules, thus reducing the operating temperature, improving the response–recovery rate and sensitivity.

### 4.4. Heterogeneous Structural Construction

Different semiconductor materials contact each other at the interface to form a heterojunction. Electrons modulate the carrier concentration, depletion layer width, and barrier height at the interface through interactions between Fermi energy levels and energy bands. This modulation changes the physical and chemical properties of the semiconductor materials, and the energy band structures at the interfaces of different types of heterojunctions change [120,121,122,123]. The composite of n- or p-type semiconductors to construct novel heterojunctions (p-n, n-n, and p-p) is an effective strategy to improve the gas-sensitive properties of the materials.

Due to the difference in the figure of merit, the majority carriers in the two materials will diffuse in opposite directions until the Fermi energy level of the whole system equilibrates. Meanwhile, a depletion layer will form at the contact interface, elevating the base resistance of the sensor. When the target gas diffuses to the surface of the material, the flow of electrons into the material system causes the material carrier concentration to increase and the resistance to decrease. The whole process makes the heterogeneous material-based sensor more sensitive to the target gas.

## 5. Cross-Sensitivity and Response Strategies

Selectivity refers to the degree to which a gas sensor is sensitive to only one specific gas in a complex atmospheric environment. Due to the working mechanism of sensitive materials, gas sensors usually respond to multiple gases, i.e., they are cross-sensitive. Reducing the cross-response of interfering gases has always been a complex and vital issue in the field of gas sensitivity [124,125].

The fabrication of composites by introducing noble metals, polymers, carbon materials, and other materials is one of the essential ways to achieve the optimization of sensor selectivity for dissolved gas detection in transformer oil [126]. Zhou et al. [127] prepared Ag-modified ZnO nanorods by employing a simple and environmentally friendly solvent–thermal method and tested the sensor’s response to ten test gases at a concentration of 100 ppm. The test gases included dissolved gases detected in transformer oil, ethylene, hydrogen, carbon monoxide, ethanol, acetone, methanol, formaldehyde, benzene, and toluene. The results showed that the response of the sensor based on 3 at% Ag-ZnO to C_2_H_2_ is much higher than that of the other nine gases and that Ag doping with the noble metal significantly improves the selectivity of the sensor.

Theoretical modeling and simulation calculation analysis can reveal the changing law of materials in the reaction process at the microscopic level and guide the design of sensitive materials for sensors, while complementing the sensitivity mechanism of gas selectivity. Jiang et al. [128] employed DFT to simulate the adsorption performance, sensitivity, and electron-leaping behavior of two-dimensional nanomaterials (Au-MoS_2_) in response to different gases dissolved in transformer oil by simulating the microscopic parameters, such as the atomic configuration, the energy band structure, the electronic density of states, the charge layout and charge transfer of the materials. The results showed that the system material has good sensing performance for C_2_H_2_, C_2_H_4,_ and C_2_H_6_, exhibiting a unique response to C_2_H_2_. In addition, the Au-MoS_2_ monolayer film has good selectivity for different oil-dissolved gases.

The pattern recognition method is a computational approach to feature extraction, analysis, and sample categorization of response data from sensors or arrays in atmospheric environments to achieve the identification of gas species and concentrations. [129,130]. A large number of studies have demonstrated that pattern recognition and algorithmic analysis can be an effective method to improve the selectivity of gas-sensitive systems [131,132,133,134,135,136]. Tang et al. [137] used functionalized modified carbon nanotube materials to prepare eight different sensors to form a sensor array, tested their gas-sensing properties in single and mixed gas environments, and combined them with DBN-DNN algorithms to achieve the qualitative recognition and quantitative analysis of the sensor arrays for mixed gases such as H_2_, CO, and C_2_H_2_.

## 6. Summary and Outlook

The DGA technique is considered to be one of the most convenient and effective methods for transformer fault diagnosis. Chemiresistive gas sensors have become the mainstream focus of gas sensor research due to their excellent performance and development potential. Based on the basic principles of chemiresistive gas sensors, this review highlights recent advances in gas sensors for the detection of dissolved gases (including H_2_, CH_4,_ C_2_H_6_, C_2_H_4_, CO and CO_2_) in transformer oil. The methods of material synthesis and their sensitivity characteristics are highlighted. Numerous experimental data show that adjusting the particle size and morphology of the sensing materials, as well as doping with noble metals, are critical strategies for improving the performance of chemiresistive gas sensors.

Although there has been a considerable amount of excellent research on gas sensors for the DGA technique, they are still in the process of being developed for industrial sensor applications. The main requirements of these sensors for practical industrial applications are as follows:

1. **Low limit of detection**: The concentration of dissolved gases in transformer oil typically requires sensors with a low LOD. Sensitivity can be enhanced by selecting suitable sensitive materials, optimizing their morphology and structure, incorporating appropriate dopants, and constructing heterojunctions. Currently, gas sensor technology is mature for detecting dissolved H_2_ and CO, with considerable research available for C_2_H_2_ and C_2_H_4_ detection. The detection of CH_4_ and C_2_H_6_ is still being explored. Due to the inert nature of CO_2_, achieving high sensitivity for its detection remains challenging with chemiresistive gas sensors.
2. **Low power consumption**: Reducing power consumption is a crucial development direction for chemiresistive gas sensors. MOS gas sensors typically require high temperatures to achieve good sensitivity, leading to high power consumption. Given the need to detect seven characteristic gases for transformer fault diagnostics, the number of required sensors increases, compounding the power consumption issue. Future advancements must focus on applying MEMS structures to reduce power consumption or on developing high-performance materials that operate effectively at low or ambient temperatures.
3. **Miniaturization and integration:** The miniaturization and integration of sensors or probes are essential for the detection of dissolved gases in transformer oil. The limited amount of dissolved gases collected by the oil/gas separator and the constraints of actual operating conditions limit the size and application of sensor probes. Therefore, a compact and integrated sensor design is essential for practical applications.

At the same time, there are still some key issues that need to be addressed in the development of more practical gas sensor devices.

1. **Unclear sensitization mechanisms:** Although different gas-sensing mechanisms and models have been proposed and developed, it is still difficult to determine certain reactions between different gas–solid interfaces, including active sites, intermediates, chemical reaction pathways, etc. More quantitative analyses at the physical and chemical levels, combining computer simulations with experimental validation, are needed to explore the factors that play a dominant role in gas sensors, among the many influencing factors.
2. **Humidity and temperature interferences:** In general, high humidity reduces the sensitivity of most gas sensors, resulting in invalid detection information. Changes in the ambient temperature can also introduce errors in the sensor.
3. **Sensor drift problem:** resistance drift and response degradation are common over long periods of operation, and this instability limits the long-term operation of gas sensors, making subsequent circuit integration difficult.
4. **Cross-sensitivity:** Cross-sensitivity poses a significant challenge, requiring highly selective sensors, integrated sensor arrays, and advanced AI algorithms to enhance system recognition capabilities. The future trend in transformer fault diagnosis involves the fusion of multiple sensors for online monitoring. These advancements will be crucial in improving the accuracy and reliability of transformer fault diagnostics.

Excellent sensing materials continue to be in high demand to address the above issues. Highly sensitive, selective, and stable sensing layers will reduce the difficulty of designing and testing circuits. In addition, reliable sensing through sophisticated corrections is expected to be further enabled by sensor arrays and post-processing of data through analysis and algorithms. With the development of sensing technologies related to sensing materials and devices, DGA is poised to make significant breakthroughs in the future.

## Figures and Tables

**Table 1:** Principle gases and associated fault types.

Fault Types	Main Gas	Secondary Gas
Oil thermal	CH_4_, C_2_H_4_	H_2_, C_2_H_6_
Oil and paper thermal	CH_4_, C_2_H_4_, CO, CO_2_	H_2_, C_2_H_6_
Partial discharge in oil paper insulation	H_2_, CH_4_, CO	C_2_H_2_, C_2_H_6_, CO_2_
Spark discharge in oil	H_2_, C_2_H_2_	
Arc in oil	H_2_, C_2_H_2_	CH_4_, C_2_H_4_, C_2_H_6_
Arc in oil and paper	H_2_, C_2_H_2_, CO, CO_2_	CH_4_, C_2_H_4_, C_2_H_6_

**Table 2:** Gas concentration ranges for the multi-component online monitoring device.

Characteristic Gas	Concentration (ppm)
H_2_	2–2000
C_2_H_2_	0.5–1000
CH_4_, C_2_H_4_, C_2_H_6_	0.5–1000
CO	25–5000
CO_2_	25–15,000

## Data Availability

No new data were created or analyzed in this study.
